# Preclinical evaluation of uPAR-ICG-FVIOs for dual-mode imaging and magnetic hyperthermia therapy in pancreatic cancer

**DOI:** 10.3389/fphar.2025.1681718

**Published:** 2025-11-28

**Authors:** Tao Luo, Kelan Zhang, Chong Wu, Xiaolong Wu, Haojie Yang, Zhigang Chen, Fen Jiang

**Affiliations:** 1 Department of Hepatobiliary Surgery, Changde Hospital, Xiangya School of Medicine, Central South University (The First People’s Hospital of Changde City), Changde, China; 2 Department of Pharmacology, Inje University College of Medicine, Busan, Republic of Korea

**Keywords:** pancreatic cancer, magnetic field therapy, local hyperthermia, magnetic iron, nanoparticles, fluorescence imaging, magnetic resonance imaging, molecular imaging

## Abstract

**Background:**

This study presents a novel targeted nanomedicine for pancreatic cancer imaging and local magnetic hyperthermia therapy (MHT): urokinase plasminogen activator receptor (uPAR)-targeted, indocyanine green (ICG)-conjugated ferrimagnetic vortex iron oxide (FVIOs) nanorings (u-I-FVIOs). uPAR is a cancer-selective membrane protein, ICG is a clinically approved near-infrared (NIR) dye, and FVIOs are well-characterized nanorings with high efficiency in heat conversion under alternating magnetic field (AMF).

**Methods & Results:**

We systematically evaluated the physicochemical and biological properties of u-I-FVIOs and demonstrated their tumor targeting capacity and AMF-dependent cancer cytotoxicity. Following intravenous (I.V.) administration, u-I-FVIOs produced robust fluorescence and MRI signals in tumors, achieving a tumor-to-background ratio of 3.5–4.5 at 12–24 h post-injection, compared with 2.5–3.0 for I-FVIOs and 1.5–2.5 for the ICG group. In a PANC-1 subcutaneous pancreatic tumor mouse model, animals received one of four treatments: Blank, Blank + AMF, u-I-FVIOs, or u-I-FVIOs + AMF. The Blank and u-I-FVIOs were administered intratumorally (I.T.), AMF exposure was applied for 600 s after the I.T. injections. u-I-FVIOs + AMF resulted in near-complete tumor regression (tumor suppression rate: 93%; mixed-effects model: *P* = 0.0001) and significantly prolonged survival (Log-rank test: HR = 0.12, *P* = 0.009) compared to the Blank control group. In contrast, the u-I-FVIOs–only group showed no antitumor effect or survival benefit. Notably, no systemic toxicity was observed in either u-I-FVIOs treatment group.

**Conclusion:**

This study presents the first theranostic applications of u-I-FVIOs, highlighting their potential as a dual-mode imaging and targeted MHT agent for pancreatic cancer.

## Graphical Abstract

**Figure:**
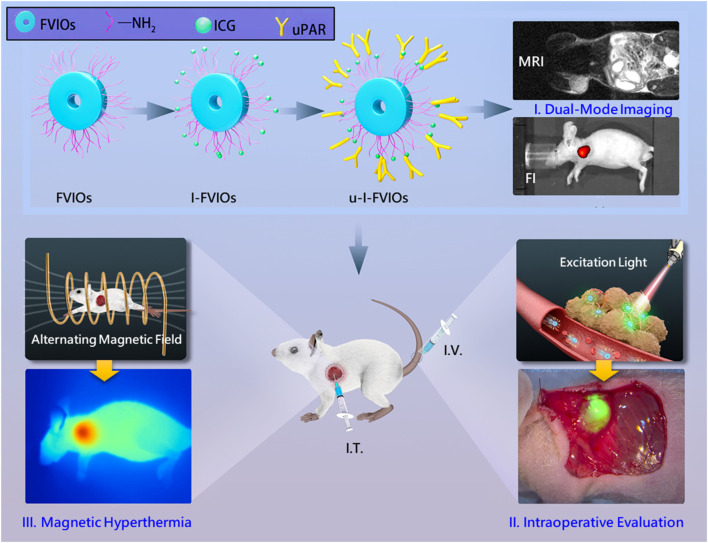
This translational study evaluates a novel nanomedicine—uPAR-targeted, ICG-conjugated FVIOs nanorings, termed u-I-FVIOs—and demonstrates its three potential applications: (I) dual-mode imaging via magnetic resonance imaging (MRI) and near-infrared (NIR) fluorescence; (II) intraoperative margin assessment; and (III) imaging-guided magnetic hyperthermia therapy (MHT).

## 1 Introduction

Pancreatic cancer remains one of the most lethal and difficult-to-treat malignancies. Magnetic hyperthermia therapy (MHT) is a cancer treatment that has recently regained momentum. It utilizes magnetic nanoparticles (MNPs) to generate localized heat when exposed to an alternating magnetic field (AMF) (Gavilan et al., 2021). MNPs for MHT are typically composed of iron oxide and injected directly into tumors, where they respond to the AMF by vibrating and converting electromagnetic energy into heat. This heat—usually in the range of 43–47 °C—selectively destroys cancer cells, which are more heat-sensitive than normal cells (Imashiro et al., 2021); moreover, MHT can enhance the efficacy of other therapies, such as chemotherapy and radiotherapy, by promoting apoptosis (Szwed and Marczak, 2024). MHT is a non-invasive treatment modality that offers deeper tissue penetration compared to other thermotherapy techniques like laser ablation, owing to the superior penetration capability of AMF (Beik et al., 2016).

Continuous efforts have been made to advance the clinical translation of MHT. In 2010, NanoTherm—a superparamagnetic iron oxide nanoparticle (SPION)-based MHT product—was approved in Germany for the treatment of brain cancer (Maier-Hauff et al., 2011). This was followed by the approval of several other SPION-based products by the U.S. Food and Drug Administration (FDA) as contrast agents for magnetic resonance imaging (MRI) (Vallabani et al., 2019). However, the application of SPIONs in cancer treatment remains limited due to several challenges such as rapid systemic clearance and low retention in tumor tissue (Bakhtiary et al., 2016).

Various strategies were adopted to overcome the current issues. For example, peptides (Du et al., 2019) or proteins (Ngen et al., 2019) that can selectively bind to cancer cells were conjugated to the drug surface to increase nanoparticles’ cancer targeting capacity. Near-infrared (NIR) fluorescent dyes were conjugated to nanoparticles to expand the drug substance’s application for fluorescence imaging (He et al., 2010). Different shapes of magnetic nanoclusters (e.g., hexagon-shaped) were explored to overcome the relatively low heating efficiency of traditional iron oxide nanoparticles (Albarqi et al., 2019).

Previously, our colleagues developed a nanotherapeutic strategy named ferrimagnetic vortex-domain nanorings (FVIOs), and this nanomedicine forms a stable magnetic sol with excellent magnetic susceptibility and magnetization (Liu et al., 2015). In the same study, FVIOs were directly compared with Resovist (a clinically approved SPION contrast agent in the EU and United States): (i) FVIOs exhibited substantially higher AMF-induced heat conversion efficiency than Resovist, with specific absorption rate (SAR) values of 2213 vs. 106 W/g (under identical AMF strength), and intrinsic loss power (ILP) values of 4.52 vs. 0.21 nH·m^2^·kg^-1^, respectively. SAR and ILP are distinct parameters for quantifying AMF-to-heat conversion efficiency: SAR depends on experimental conditions such as nanoparticle concentration, medium, and AMF strength, whereas ILP reflects the nanoparticles’ intrinsic heating efficiency independent of AMF strength. (ii) FVIOs showed a smaller reduction in SAR when the dispersion medium was changed from water to agarose gel (25% vs. 50%), suggesting that their energy conversion efficiency is less susceptible to loss after nanoparticle uptake by tumor tissue or cells. (iii) FVIOs were larger than Resovist (130 vs. 60 nm), which facilitate improved tumor accumulation through the enhanced permeability and retention (EPR) effect.

In animal models, FVIOs demonstrated potent anti-tumor effects in combination of PD-L1 inhibitors (Liu et al., 2019). And showed potential for magnetic resonance imaging (MRI) applications, particularly T2-weighted imaging (Bao et al., 2022). In the present study, we further engineered FVIOs by conjugating them with the near-infrared (NIR) dye indocyanine green (ICG) and a urokinase plasminogen activator receptor (uPAR)-targeting ligand via amine (-NH_2_) linkages. The resulting construct, termed uPAR-ICG-FVIOs (u-I-FVIOs), was designed for dual-mode cancer imaging and image-guided MHT.

uPAR is a glycosylphosphatidylinositol (GPI)-anchored membrane receptor that binds its ligand, urokinase plasminogen activator (uPA), facilitating the conversion of plasminogen into plasmin and thereby promoting proteolytic activity. uPAR is overexpressed in both the parenchyma and invasive margins of pancreatic cancer tissues, while its expression in normal pancreatic tissue is minimal to undetectable (Metrangolo et al., 2021). In a previous study, uPAR demonstrated the highest accuracy among 29 biomarkers in distinguishing pancreatic ductal adenocarcinoma (PDAC) from chronic pancreatitis (Chen et al., 2007). Furthermore, uPAR-targeted magnetic iron oxide nanoparticles have exhibited enhanced tumor-specific retention and distribution following I.V. injection (Yang et al., 2009). It’s worth noting that uPAR’s natural ligand, uPA, is also overexpressed in tumors (Mahmood et al., 2018). Unlike uPAR being a membrane protein, uPA is a secreted protein that is distributed throughout the extracellular matrix (ECM) and body fluids. Therefore, targeting uPAR may result in more spatially confined effects at the tumor site, which is advantageous for imaging and therapeutic applications.

In addition, ICG, an amphiphilic cyanine dye, was incorporated into the FVIOs for fluorescence imaging. ICG is a near-infrared (NIR) fluorescent agent approved by the U.S. Food and Drug Administration (FDA) for diagnostic imaging of blood flow in human. Recent years have witnessed a fast-growing off-label use of ICG-mediated fluorescence imaging, for real-time intraoperative identification of tumors and metastatic lymph nodes, as well as for guiding minimally invasive tumor resection (Dalli et al., 2021; Feng et al., 2023; van Dam et al., 2024). ICG provides moderate tissue penetration (typically up to 5–10 mm) and relatively low background autofluorescence but suffers from a short circulation time and poor cancer cell specificity. Therefore, in recent years, ICG has been conjugated to nanoparticles to overcome these limitations (Chauhan et al., 2023).

This study aimed to comprehensively evaluate the physicochemical properties, biological behavior, imaging capabilities, and therapeutic potential of u-I-FVIOs as a local MHT in pancreatic cancer through a series of *in vitro* and *in vivo* assessments.

## 2 Materials and methods

### 2.1 Chemicals and materials

FeCl_3_, NaH_2_PO_4_, Na_2_SO_4_, H_2_SO_4_, HNO_3_, and NH_4_H_2_PO_4_ were purchased from Sinopharm Chemical Reagent Co., Ltd. (Shanghai, China). The N-hydroxysuccinimide ester derivative of indocyanine green (ICG-NHS) was purchased from Goryo Chemical, Inc (Sapporo, Japan). 1-(3-Dimethylaminopropyl)-3-ethylcarbodiimide hydrochloride (EDC) and NHS were from Adamas Reagent, Ltd (Shanghai, China). DSPE-PEG5000-NH_2_ was purchased from Xi’an Ruixi Biological Technology Co., Ltd (Xi’an, China). The rabbit polyclonal anti-uPA Receptor/uPAR IgG antibody was obtained from Abcam (ab103791, Cambridge, United Kingdom). All chemicals were of reagent grade and used without further purification. Millipore Milli-Q grade deionized water was used throughout the study.

Dulbecco’s Modified Eagle Medium (DMEM), Roswell Park Memorial Institute 1,640 Medium (RPMI-1640), and fetal based from Gibco, Life Technologies (Stockholm, Sweden). Phosphate buffer saline (PBS), trypsin, EDTA and penicillin-streptomycin solution were obtained from M&C Gene Technology, Ltd (Beijing, China).

### 2.2 Cell lines and cell culture

The human pancreatic cancer cell line SW1990 was purchased from PerkinElmer (Illinois, United States), while the PANC-1 cell line was obtained from GeneChem Co., Ltd (Shanghai, China). The normal human pancreatic duct epithelial cell line HPED6-C7 was acquired from Shanghai Zeye Biotechnology Co., Ltd (Shanghai, China). All cells were cultured at 37 °C in a 5% CO_2_ atmosphere. PANC-1 was grown in DMEM containing 10% FBS, 1% penicillin-streptomycin solution while SW1990 and HPDE6-C7 were cultured in RPMI-1640, supplemented with 10% FBS and 1% penicillin-streptomycin solution. All experiments were performed using cells in the logarithmic growth phase.

### 2.3 Synthesis of FVIOs suspension

Ferrimagnetic vortex-domain Fe_3_O_4_ nanorings were prepared as previously reported (Liu et al., 2015). Briefly, in a typical experimental procedure, specific amounts of FeCl_3_, NaH_2_PO_4_, and Na_2_SO_4_ aqueous solutions were mixed, and distilled water was added to adjust the final volume to 80 mL. The final concentrations of FeCl_3_, NaH_2_PO_4_, and Na_2_SO_4_ were 0.02, 1.8 × 10^−4^, and 5.5 × 10^−4^ mol/L, respectively. After vigorous stirring for 10 min, the mixture was transferred into a 100 mL Teflon-lined stainless-steel autoclave and subjected to hydrothermal treatment at 220 °C for 48 h. The resulting precipitate was collected by centrifugation, washed with distilled water and absolute ethanol, and dried under vacuum at 80 °C. A single batch yielded over 120 mg of α-Fe_2_O_3_ nanorings.

Varying the concentration of NH_4_H_2_PO_4_ and Na_2_SO_4_ results in α-Fe_2_O_3_ nanorings and nano-tubes with diverse sizes and surface morphologies. To investigate the role of NH_4_H_2_PO_4_ and Na_2_SO_4_, the synthesis was repeated using either NH_4_H_2_PO_4_ or Na_2_SO_4_ individually, while maintaining all other reaction conditions. Fe_3_O_4_ nanorings were then obtained via a reduction process using the α-Fe_2_O_3_ products as precursors. The dried α-Fe_2_O_3_ powders were annealed in a furnace at 360 °C under a continuous hydrogen/argon gas flow, H_2_/(H_2_ + Ar) = 8/100, for 5 h, followed by cooling to room temperature under hydrogen flow. Magnetite (Fe_3_O_4_) nanorings were synthesized by first hydrothermally growing α-Fe_2_O_3_ nanorings, followed by hydrogen reduction using the α-Fe_2_O_3_ as a template.

The FVIOs suspension was prepared by modifying the Fe_3_O_4_ nanoring surface with DSPE-PEG5000-NH_2_ (Gao et al., 2019). Specifically, Fe_3_O_4_ nanorings were dispersed in water at 1 mg/mL by ultrasonication. Then, 80 mg of DSPE-PEG5000-NH_2_ was added, and the mixture was stirred under reflux for 4 h with a constant argon flow. The suspension was washed three times with water using magnetic decantation, and the FVIOs were re-dispersed in water at a final Fe_3_O_4_ concentration of 1 mg/mL.

### 2.4 Synthesis of ICG-FVIOs (I-FVIOs)

To conjugate FVIOs with ICG for I-FVIOs, ICG-NHS (300 μg) and FVIOs (1 mg) were dissolved in deionized water. The mixture was shaken at 4 °C overnight and then centrifuged at 14,000 rpm for 15 min. The precipitate was lyophilized to obtain I-FVIOs, while the supernatant was collected to determine the ICG loading efficiency using a UV-Vis spectrophotometer (UV-2450, Shimadzu, Japan). The loading efficiency (%) was calculated using the following formula:

$$\text{Loading efficiency of ICG }\left(\%\right)=\frac{\text{Drug}\;\text{input}\;\left(\mu g\right)–\;\text{Drug}\;\text{in}\;\text{supernatant}\;\left(\mu g\right)}{\text{Drug}\;\text{input}\;\left(\mu g\right)}\times 100$$

### 2.5 Synthesis of uPAR-ICG-FVIOs (u-I-FVIOs)

To further conjugate uPAR antibodies (uPAR-Ab) to I-FVIOs and obtain u-I-FVIOs, I-FVIOs dispersed in PBS (pH 6.0, 5 mL) were activated with EDC (0.02 g) and NHS (0.02 g) at 0 °C for 2 h (Roshni et al., 2019). Following activation, uPAR-Ab (100 μg) was added, and the mixture was stirred for 24 h. The resulting solution was then dialyzed and lyophilized to obtain u-I-FVIOs. The final product was water-soluble, exhibited minimal aggregation under normal conditions, and could be rapidly aggregated in the presence of an external magnetic field.

### 2.6 Characterization

The particle size and morphology of u-I-FVIOs were examined using a scanning electron microscope (SEM; model 4,300, Hitachi, Tokyo, Japan). Dynamic light scattering (DLS) was employed to measure the hydrodynamic diameter using a Malvern Zetasizer (ZEN 3600, United Kingdom). Fluorescence excitation and emission spectra were measured using a fluorescence spectrofluorometer (F-7000, Hitachi, Japan). The stability of u-I-FVIOs (50 μg) in fetal bovine serum (FBS, 1 mL) was evaluated by monitoring optical absorbance at 781 nm with the spectrophotometer at multiple time points (8, 16, 24, 36, and 48 h). Fluorescence intensity in mice administered with varying concentrations of u-I-FVIOs (9.375, 18.75, 37.5, 75, 150, 300, and 600 μg/mL) was quantified using the IVIS® Spectrum optical imaging system (PerkinElmer, Illinois, United States). The transverse relaxation rate (1/T2, s^-1^) of u-I-FVIOs at various concentrations (0.042, 0.084, 0.167, 0.335, 0.670, and 1.339 mM) was determined using an MRI system (M3, ASPECT, Israel). All samples for MRI relaxivity studies were prepared in 1.5% agar gel. The magnetic hyperthermia effects were evaluated using an electromagnetic induction heater (EASYHEAT-5060, Ambrell, United States). PBS solution containing u-I-FVIOs, I-FVIOs, FVIOs, uPAR-Ab (all at 50 μg/mL), and free ICG (16.7 μg/mL), as well as u-I-FVIOs at varying concentrations (0, 25, 50, 100, and 200 μg/mL), were exposed to an AMF (495 kHz, 220 Oe) for 600 s. Temperature changes were recorded at 1-s intervals using a fiber optic temperature probe (Neoptix, Quebec, Canada). Infrared thermal images of the samples were captured using a thermal imaging camera (FLUKE Ti25, United States).

### 2.7 Cytotoxicity assay

The cytotoxicity of the u-I-FVIOs was assessed using the Cell Counting Kit-8 (CCK-8, Solarbio, Santa Clarita, United States). PANC-1 and SW1990 cells were seeded in 96-well plates at a density of 1 × 10^4^ cells per well and incubated for 24 h. The culture medium was then replaced with fresh medium containing various concentrations of u-I-FVIOs, I-FVIOs, and FVIOs (10, 25, 50, 100, 200, 300, 400 μg/mL), along with corresponding concentrations of free ICG (3.34,8.35, 16.7, 33.4, 66.8, 100.2, 133.6 μg/mL). Subsequently, the cells were incubated with CCK-8 for an additional 1 h. Absorbance was measured using a microplate reader (Synergy HT, BioTek, Winooski, United States). All experiments were performed in triplicate.

### 2.8 *In vitro* tumor targeting

To evaluate the cell-targeting capacity and cellular uptake of u-I-FVIOs, pancreatic cancer cells (PANC-1 and SW 1990) and normal pancreatic duct epithelial cells (HPDE6-C7) were seeded in live-cell fluorescence microscopy (LCFM) culture dishes and preincubated for 4 h with u-I-FVIOs (100 μg/mL), I-FVIOs (100 μg/mL), or free ICG (33.4 μg/mL) in FBS-free culture medium. The cells were then washed three times with PBS. Cellular uptake was visualized using an inverted fluorescence microscope (M205FA, Leica Microsystems, Wetzlar, Germany) and quantified based on fluorescence intensity within regions of interest (ROI) using ImageJ 2 software.

### 2.9 Determination of uPAR expression

uPAR protein expression was analyzed by Western blot (WB) as previously described (Wei et al., 2017). Human pancreatic cancer cells (PANC-1, SW 1990), and normal pancreatic duct epithelial cells (HPDE6-C7) were seeded in 6-well plates and incubated for 24 h. After centrifugation to remove the supernatant, cell pellets were lysed in RIPA buffer and incubated on ice for 30 min, followed by denaturation at 100 °C for 5 min. For polyacrylamide gel electrophoresis (PAGE), a 12% resolving gel and a 5% stacking gel were used, with each sample (50 µg) loaded per lane. Electrophoresis was performed at 80V for stacking and 120V for separation until bromophenol blue reached the bottom of the gel. Proteins were transferred onto a polyvinylidene fluoride (PVDF) membrane using a wet transfer system at a constant current of 300 mA for 2 h. The membrane was blocked for 1 h in 1% BSA or 5% milk, followed by overnight incubation at 4 °C with the primary antibody. After multiple washes with TBST, the membrane was incubated with an HRP-conjugated secondary antibody (1:5,000 dilution) for 50 min at room temperature. Protein detection was performed using enhanced chemiluminescence (ECL), and images were captured on X-ray film. Band intensity was quantified using Quantity One v.4.6.2 software.

For immunohistochemistry (IHC) (Jin et al., 2019), pancreatic tumor tissue sections were deparaffinized, rehydrated, and treated with 3% H_2_O_2_ for 15 min at room temperature to block endogenous peroxidase activity. After blocking with 5% BSA for 30 min, sections were incubated overnight at 4 °C with the primary antibody. The next day, the recommended secondary antibody working solution was applied and incubated at 37 °C for 30 min. Color development was performed using a diaminobenzidine (DAB) substrate kit (Servicebio, Wuhan, China). Finally, the sections were dehydrated, mounted with neutral gum, and examined under a microscope.

### 2.10 *In vitro* magnetic hyperthermia effects

To evaluate the *in vitro* magnetic hyperthermia effect of u-I-FVIOs, the procedure was conducted in accordance with the recommendations of Spirou et al. (2018). Pancreatic cancer cells (PANC-1 and SW 1990) were cultured in 6-well plates at a density 1 × 10^5^ cells per well. After 24 h of incubation, the culture medium was replaced with fresh medium containing various concentrations of u-I-FVIOs (25, 50, 100, and 150 μg/mL). The cells were then incubated for an additional 24 h before harvesting. The collected cell suspension was transferred into plastic tubes and exposed to an AMF (495 kHz, 220 Oe) for 600 s. Untreated cells, with or without AMF exposure, were served as controls. Following treatment, the cells were re-seeded into 96 well plates and incubated for an additional 4 h before performing the CCK-8 assay.

To further assess the cell apoptosis caused by magnetic hyperthermia, a calcein-AM and propidium iodide (PI) staining method was employed. After incubation with the staining solution for 15 min, the cells were observed using an inverted fluorescence microscope. AM, a vital dye, stains live cells green, while PI selectively stains cells with compromised membrane integrity, appearing red under fluorescence imaging.

### 2.11 Experimental animals

This study involved five animal experiments using BALB/c male mice (4–6 weeks old) obtained from Beijing Vital River Laboratory Animal Technology Co., Ltd. (Beijing, China). All experimental protocols were approved by the Institutional Animal Care and Ethics Committee of the Molecular Imaging Laboratory at the Institute of Automation, Chinese Academy of Sciences, and all procedures were conducted in accordance with the approved guidelines.

### 2.12 Fluorescence imaging and MRI

In this animal experiment, a human pancreatic cancer-bearing model was established by injecting PANC-1 cells (1 × 10^7^) subcutaneously into the right flank of each mouse. Tumor nodules were allowed to grow to 500 mm^3^ before the mice were grouped (n = 3 per group) based on tumor volume. Tumor volume was calculated based on tumor length and width using the equation: Volume (V) = Length × Width × Width/2. Each group was then randomly assigned to receive a single I.V. injection of u-I-FVIOs (300 μg/mL, 200 μL), I-FVIOs (300 μg/mL, 200 μL), or ICG (100.2 μg/mL, 200 μL). Optical images were acquired at various time points (1, 2, 4, 8, 12, 18, 24, and 48 h) post-injection using the IVIS® spectrum imaging system. In a separate study, the mice were sacrificed 24 h post-I.V. injection, and tumor tissues along with major organs (heart, liver, spleen, pancreas, lung, kidney and intestine) were carefully dissected for fluorescence imaging. Fluorescence intensities were analyzed using Living Imaging® software (Ver 4.4).

To assess the MRI imaging performance of u-I-FVIOs, PANC-1 pancreatic tumor-bearing mice were I.V. injected with u-I-FVIOs (300 μg/mL, 200 μL), I-FVIOs (300 μg/mL, 200 μL), or PBS (200 μL). MRI scans were acquired at various time points before and after injection (12, 24, and 48 h). To visualize iron deposition in tumor tissues, Perls’ Prussian blue staining was performed on 5 μm-thick formalin-fixed, paraffin-embedded tumor sections, following the method described by Tseng et al. (2009).

### 2.13 Intraoperative margin evaluation

To evaluate the intraoperative margin assessment capability of u-I-FVIOs, PANC-1 pancreatic tumor-bearing mice were anesthetized and positioned in the right lateral position 18 h after I.V. injection of u-I-FVIOs (300 μg/mL, 200 μL), I-FVIOs (300 μg/mL, 200 μL), or ICG (100.2 μg/mL, 200 μL). Following a standard surgical procedure to expose the tumors, the surgical site was illuminated with a 785 nm laser at an intensity of 1.0 W/cm^2^. Fluorescence images were then captured using a custom-built fluorescence microscope.

### 2.14 *In vivo* hyperthermia therapy

To evaluate the *in vivo* magnetic hyperthermia effect of u-I-FVIOs, PANC-1 tumor-bearing mice were randomly assigned into four treatments (n = 6 per group): Blank, Blank + AMF, u-I-FVIOs, and u-I-FVIOs + AMF. The Blank and Blank + AMF groups received an intratumoral (I.T.) injection of PBS (200 μL), and the -I-FVIOs and u-I-FVIOs + AMF groups received an I.T. injection of u-I-FVIOs (1,000 μg/mL, 200 μL), administered using a 26-gauge needle. Right after the I.T. injections, mice in the Blank + AMF and u-I-FVIOs + AMF groups were exposed to an AMF (495 kHz, 220 Oe) for 600 s, on days 0 and 3. Infrared thermal images and the average temperatures of regions of interest (ROIs) were captured at a single time point*—*600 s after the I.T. injection of Blank or u-I-FVIOs, using an infrared thermal imaging camera. For each mouse, the ROI was manually delineated to encompass the entire tumor region. Tumor volume (V) and body weight were recorded every 3 days for a duration of 21 days. Tumor suppression rate (%) was calculated using equation:

$$\text{Tumor suppression rate}\left(\%\right)=\frac{V_{control}\;–\;V_{treatment}}{V_{control}}\times 100$$

Survival analysis was performed using the Kaplan-Meier method. Tumor tissues and major organs were harvested and stained with hematoxylin and eosin (H&E). Apoptosis within the tumor was further evaluated using the TUNEL assay (Servicebio, China) and visualized with an inverted microscope.

### 2.15 Statistical analysis

All statistical analyses were performed using GraphPad Prism software (Ver 9.5.1, GraphPad, SanDiego, United States). Mean values are presented as mean ± standard deviation. Group differences were assessed using one-way or two-way analysis of variance (ANOVA), as appropriate, followed by *post hoc* tests for multiple comparisons with recommended correction (e.g., Tukey’s test). Survival curves were analyzed using the Kaplan-Meier method, and differences between groups were assessed using the log-rank (Mantel–Cox) test. A *P*-value of less than 0.05 was considered statistically significant.

## 3 Results

### 3.1 uPAR was specifically expressed by malignant pancreatic cells

**FIGURE 1:**
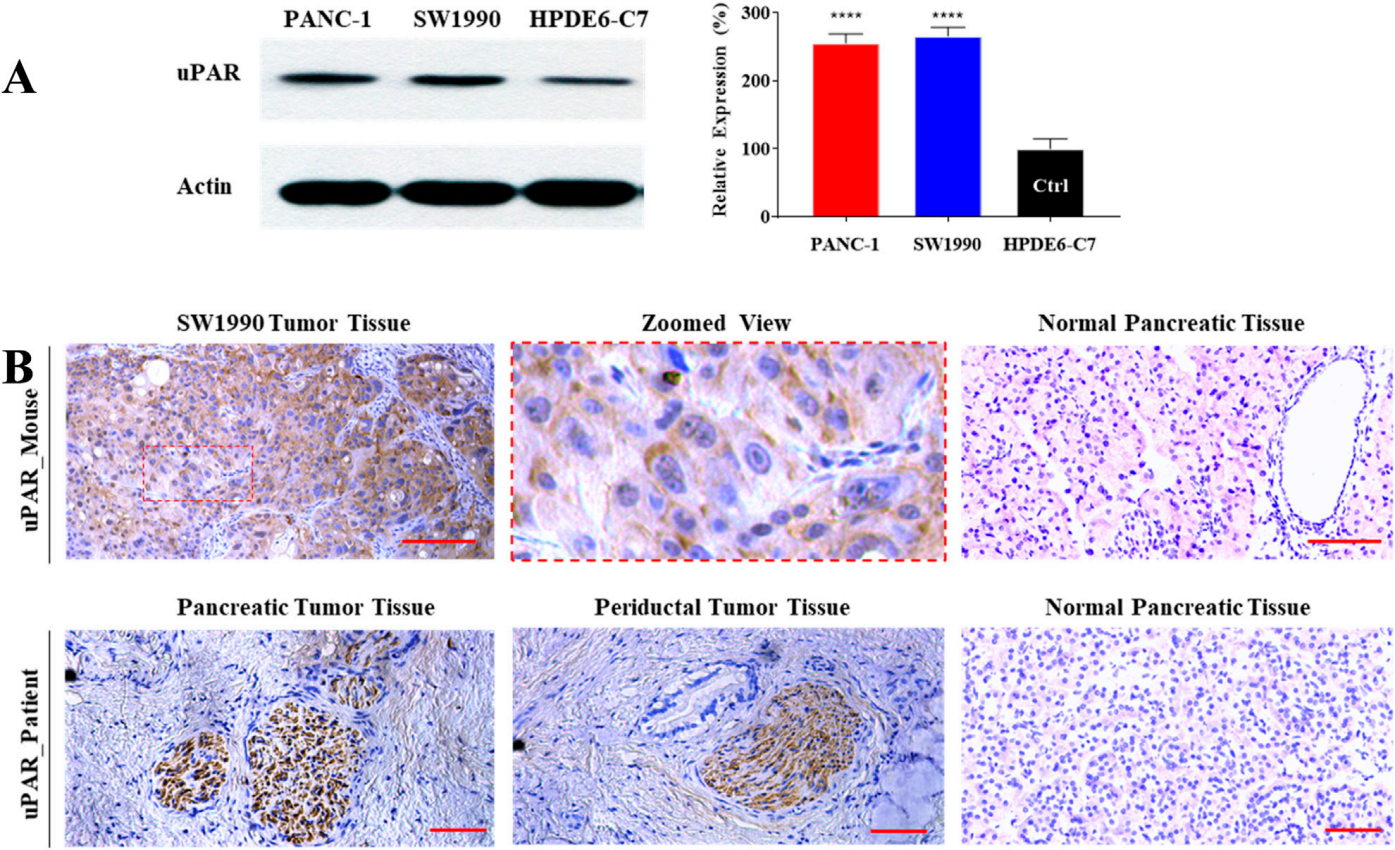
High-level expression of uPAR in human pancreatic cancer. **(A)** Western blot analysis of uPAR expression in two pancreatic cancer cell lines (PANC-1 and SW 1990) and a normal human pancreatic duct epithelial cell line (HPDE6-C7). Compared to HPDE6-C7, ****: *P* < 0.0001. **(B)** Immunohistochemical (IHC) staining of uPAR in pancreatic tumor tissue from SW1990 tumor-bearing mice (upper panel) and a pancreatic cancer patient (lower panel). uPAR-positive regions are indicated by brown chromogenic staining. Scale bar: 50 µm.

We evaluated uPAR expression at three levels: cell lines, a xenograft mouse model, and surgical specimens from a 69-year-old male pancreatic cancer patient (with written informed consent obtained prior to surgery). First, uPAR expression in two pancreatic cancer cell lines (PANC-1 and SW 1990) and a normal pancreatic duct epithelial cell line (HPDE6-C7) was measured by Western blot. As shown in Figure 1A, uPAR expression was more than 200% higher in both PDAC cell lines compared to the normal pancreatic epithelial cells (*P* < 0.0001). Second, immunohistochemistry (IHC) was conducted to assess uPAR expression in pancreatic tumor tissues. As shown in Figure 1B, clear uPAR staining was observed on the cell membrane of in SW1990-derived subcutaneous tumors in the mouse model. Elevated uPAR expression was also detected in the cytoplasm of pancreatic tumor specimens from both mice and human patients, whereas no uPAR expression was detected in the normal pancreatic tissues from either species.

### 3.2 Characterization of uPAR-ICG-FVIOs (u-I-FVIOs)

**FIGURE 2:**
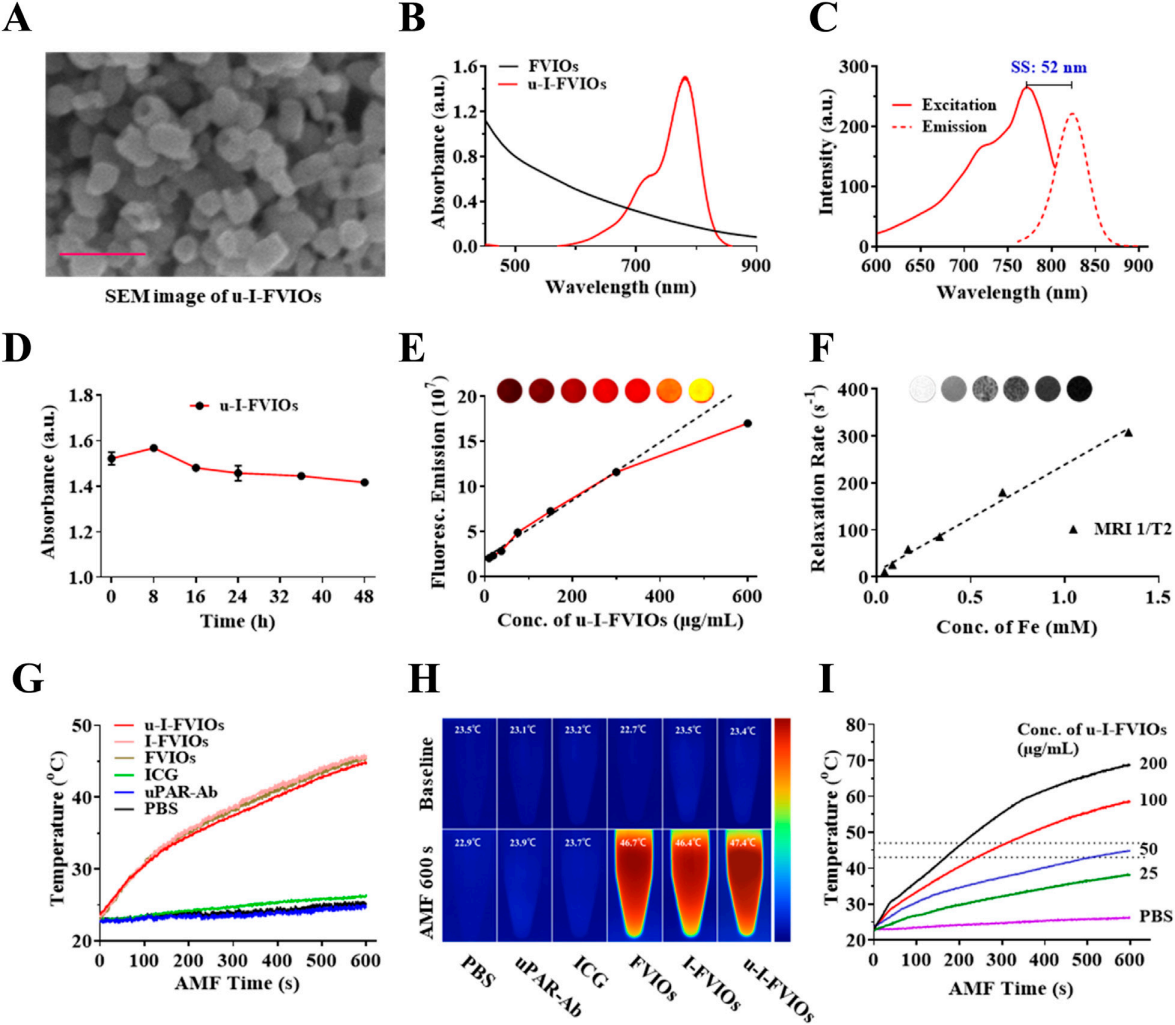
Characterization of u-I-FVIOs. **(A)** Scanning electron microscopy (SEM) image of u-I-FVIOs; scale bar: 200 nm. **(B)** UV-vis-NIR absorption spectra of unconjugated FVIOs and u-I-FVIOs. **(C)** Excitation and emission spectra of u-I-FVIOs, showing an excitation peak at 771 nm and a fluorescence emission peak at 825 nm. SS: Stokes shift, the difference between the peak excitation and emission wavelengths. **(D)** Serum stability of u-I-FVIOs at a concentration of 50 μg/mL over a period of 48 h. **(E)** Fluorescence intensity of u-I-FVIOs as a function of increasing concentration (0–600 μg/mL). Inset: fluorescence signal images acquired using an *in vivo* optical imaging system (IVIS spectrum). **(F)** T2 relaxation rate (1/T2, s^-1^) of u-I-FVIOs as a function of increasing concentration. Inset: representative T2-weighted MR images. **(G)** Temperature changes and **(H)** Corresponding near-infrared (NIR) thermal images of six different samples (50 μg/mL) under an alternating magnetic field (AMF; 495 kHz, 220 Oe) for 600 s. **(I)** Temperature changes of u-I-FVIOs at different concentrations (0, 25, 50, 100, and 200 μg/mL) under an AMF (495 kHz, 220 Oe) for 600 s. Dash lines indicate an ideal MHT temperature range of 43 °C–47 °C.

Scanning electron microscopy (SEM) imaging (Figure 2A) revealed that u-I-FVIOs were well-dispersed, exhibiting a uniform ring-shaped morphology with an average outer diameter of ∼70 nm, an inner diameter of ∼40 nm, and a height of ∼50 nm. Dynamic light scattering (DLS) analysis (Supplementary Figure S1) showed that u-I-FVIOs had a hydrodynamic diameter of approximately 150.9 nm, with a polydispersity index (PDI) of 0.145. A PDI below 0.2 indicates a monodisperse size distribution, which is considered acceptable for nanoparticle formulations. The UV-vis-NIR absorbance of the samples was measured across a wavelength range of 450–900 nm. Compared to FVIOs, u-I-FVIOs exhibited a distinct absorbance peak at 780 nm, indicating the successful conjugation of ICG (Figure 2B). The excitation and emission spectra of u-I-FVIOs (Figure 2C) showed maximum peaks at 771 nm (excitation) and 825 nm (emission), respectively, confirming that the optical properties of ICG were preserved after modification. To assess serum stability, u-I-FVIOs were suspended in 10% fetal bovine serum (FBS) to mimic blood plasma conditions. As shown in Figure 2D, the absorbance at 780 nm remained stable over 48 h, suggesting good colloidal stability. The fluorescence emission intensity of u-I-FVIOs was further evaluated across a concentration range of 9.37–600 μg/mL: as shown in Figure 2E, at concentration ≤300 μg/mL the fluorescence intensity increased linearly with concentration (Y = 331,872.07X + 19,131,343.28, *R* ^2^ = 0.9907, *P* < 0.001). At concentrations >300 μg/mL, the fluorescence intensity started to saturate. Finally, the MRI signal of u-I-FVIOs was assessed (Figure 2F): a clear concentration-dependent T2 signal reduction was observed, with a linear correlation (Y = 227.39X + 11.61, *R* ^2^ = 0.9911, *P* < 0.001).

The magnetic hyperthermia properties of the samples were further evaluated using thermal imaging under an alternating current magnetic field (AMF). Upon AMF exposure (495 kHz, 220 Oe), the temperatures of FVIOs, I-FVIOs, and u-I-FVIOs at a concentration of 50 μg/mL increased rapidly at similar rates, approaching approximately 47 °C within 600 s. In contrast, the temperatures of PBS, uPAR-Ab, and ICG samples remained largely unchanged over the same period (Figures 2G,H). Furthermore, u-I-FVIOs demonstrated a concentration-dependent magnetic hyperthermia effect, achieving a maximum temperature of 69 °C at a concentration of 200 μg/mL after 600 s of AMF exposure (Figure 2I).

### 3.3 U-I-FVIOs showed no cytotoxicity but cancer targeting *in vitro*

**FIGURE 3:**
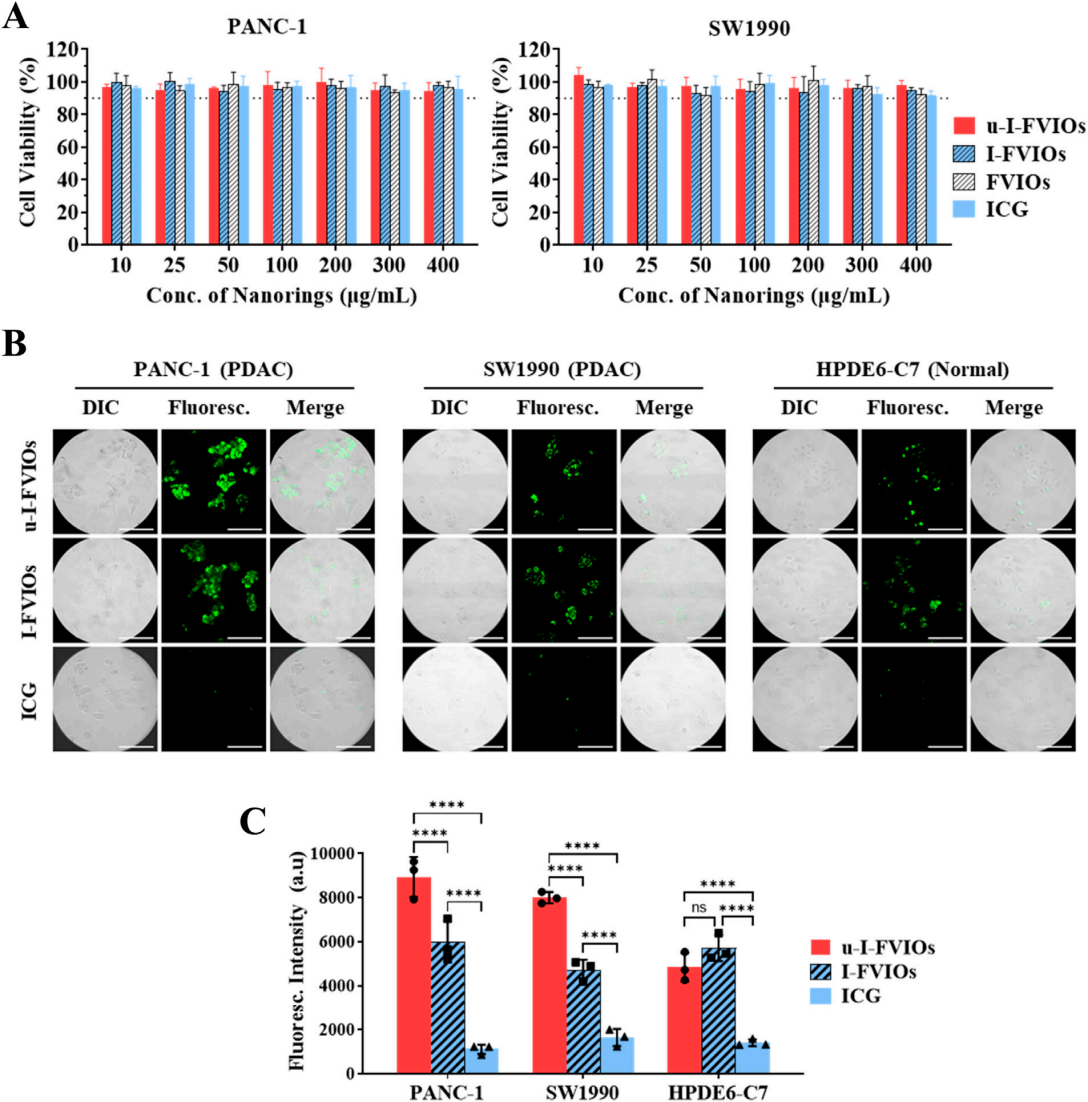
u-I-FVIOs exhibit no cytotoxicity and show strong cancer cell specificity. **(A)** Viability of PANC-1 and SW1990 cells after 1 h incubation with 10–400 μg/mL of ICG, FVIOs, I-FVIOs, or u-I-FVIOs. Dash lines: 90% viability. **(B)** Live-cell imaging showing uptake of I-FVIOs (100 μg/mL), u-I-FVIOs (100 μg/mL), or ICG (an equivalent concentration of 33.4 μg/mL), by PANC-1, SW 1990, and HPDE6-C7 cells after 4 h incubation. Scale bar: 50 μm. **(C)** Quantification of fluorescence intensity corresponding to cellular uptake of ICG, I-FVIOs, and u-I-FVIOs in PANC-1, SW 1990, and HPDE6-C7 cells. ns: *P* > 0.05; ****: *P* < 0.0001.

The cytotoxic effects of the prepared samples—including ICG, FVIOs, I-FVIOs, and u-I-FVIOs—were evaluated in pancreatic cancer cell lines (PANC-1 and SW 1990) using CCK-8 assays. As shown in Figure 3A, no significant cytotoxicity was observed across the concentration range of 10–400 μg/mL, with average cell viability remaining above 90% in all treatment groups for both cell lines.

The cancer cell targeting of ICG, I-FVIOs, and u-I-FVIOs was assessed using a live-cell fluorescence assay. As shown in Figure 3B, after a 4 h incubation with ICG, no detectable fluorescent signals were observed in either cancerous or normal cells, consistent with previous reports indicating poor cellular uptake of ICG (Qi et al., 2018). In contrast, fluorescence was detected in all 3 cell lines following incubation with I-FVIOs or u-I-FVIOs, likely reflecting a shared internalization mechanism previously reported for FVIOs (Dias et al., 2017). Notably, as shown in Figure 3C, I–U-FVIOs produced significantly stronger fluorescent signals than I-FVIOs in cancer cells—averaging 50% (*P* < 0.0001) and 70% (*P* < 0.0001) higher in PANC-1and SW1990 cells, respectively—but not in normal cells (*P* > 0.05). Moreover, the fluorescence intensity of u-I-FVIOs in PANC-1 and SW1990 cells was 1.8-fold (*P* < 0.0001) and 1.7-fold (*P* < 0.0001) higher than that in the normal cells. The *P* values in this experiment were obtained using a two-way ANOVA analysis and followed by *post hoc* Tukey’s tests for multiple comparisons with recommended correction. This suggests that specific binding to cancer-associated uPAR is a key mechanism underlying the enhanced signal intensity of u-I-FVIOs, compared to I-FVIOs.

### 3.4 *In vitro* magnetic hyperthermia studies

**FIGURE 4:**
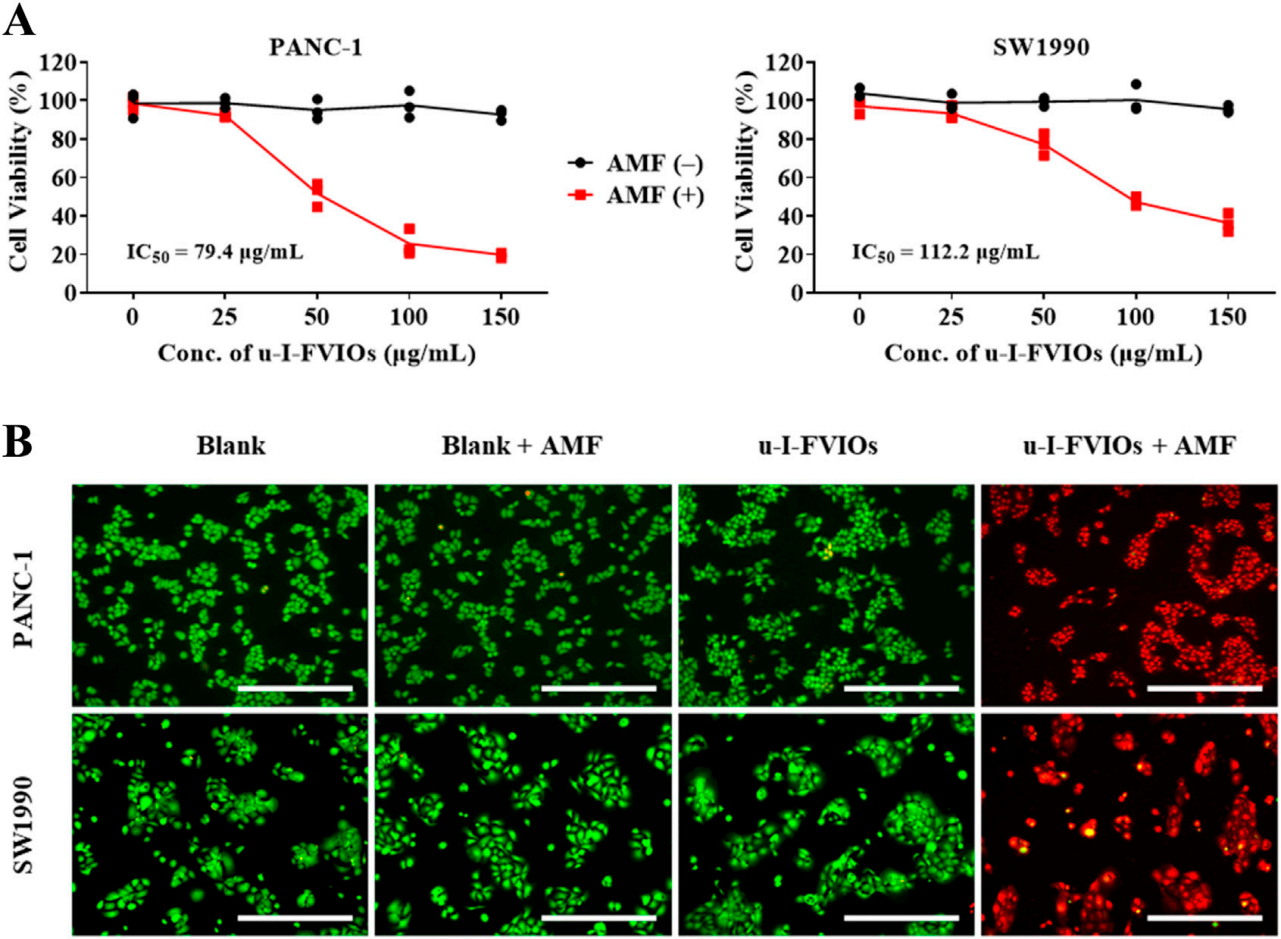
u-I-FVIOs induced magnetic hyperthermia effects in pancreatic cancer cells. **(A)** Viability of PANC-1 and SW1990 cells after 24 h incubation with 0–150 μg/mL u-I-FVIOs, with or without exposure to an alternating magnetic field (AMF) for 600 s, as assessed by CCK-8 assay. **(B)** Calcein AM/PI double staining images of PANC-1 and SW1990 cells treated with blank or 200 μg/mL u-I-FVIOs, with or without exposure to an AMF for 600 s. Green and red fluorescence indicate live and dead cells, respectively. Scale bar: 500 μm.

The magnetic hyperthermia effects of u-I-FVIOs were evaluated in pancreatic cancer cell lines. As shown in Figure 4A,I–U-FVIOs alone exhibited no cytotoxicity toward either PANC-1 or SW1990 cells in the absence of an AMF. However, following incubation with u-I-FVIOs and subsequent exposure to AMF for 600 s, both cell lines exhibited significant, dose-dependent cytotoxicity across the tested concentration range (0–150 μg/mL). The half-maximal inhibitory concentrations (IC_50_) were calculated to be 79.4 μg/mL for PANC-1 cells and 112.2 μg/mL for SW1990 cells. To further evaluate cell viability, calcein-AM (live cell stain) and propidium iodide (PI, dead cell stain) were used. As shown in Figure 4B, cells treated with 200 μg/mL u-I-FVIOs and exposed to AMF (495 kHz, 220 Oe) displayed complete cell death. In contrast, cells treated with u-I-FVIOs alone, without AMF exposure, showed minimal cytotoxicity.

### 3.5 Distribution of u-I-FVIOs *in vivo*

To investigate the *in vivo* biodistribution of u-I-FVIOs, PANC-1 subcutaneous tumor-bearing mice were divided into three groups and administered u-I-FVIOs, I-FVIOs, or ICG via I.V. injection through the tail vein. Fluorescence images were acquired using the IVIS imaging system at multiple time points (1, 2, 4, 8, 12, 18, 24, and 48 h) post-injection.

**FIGURE 5:**
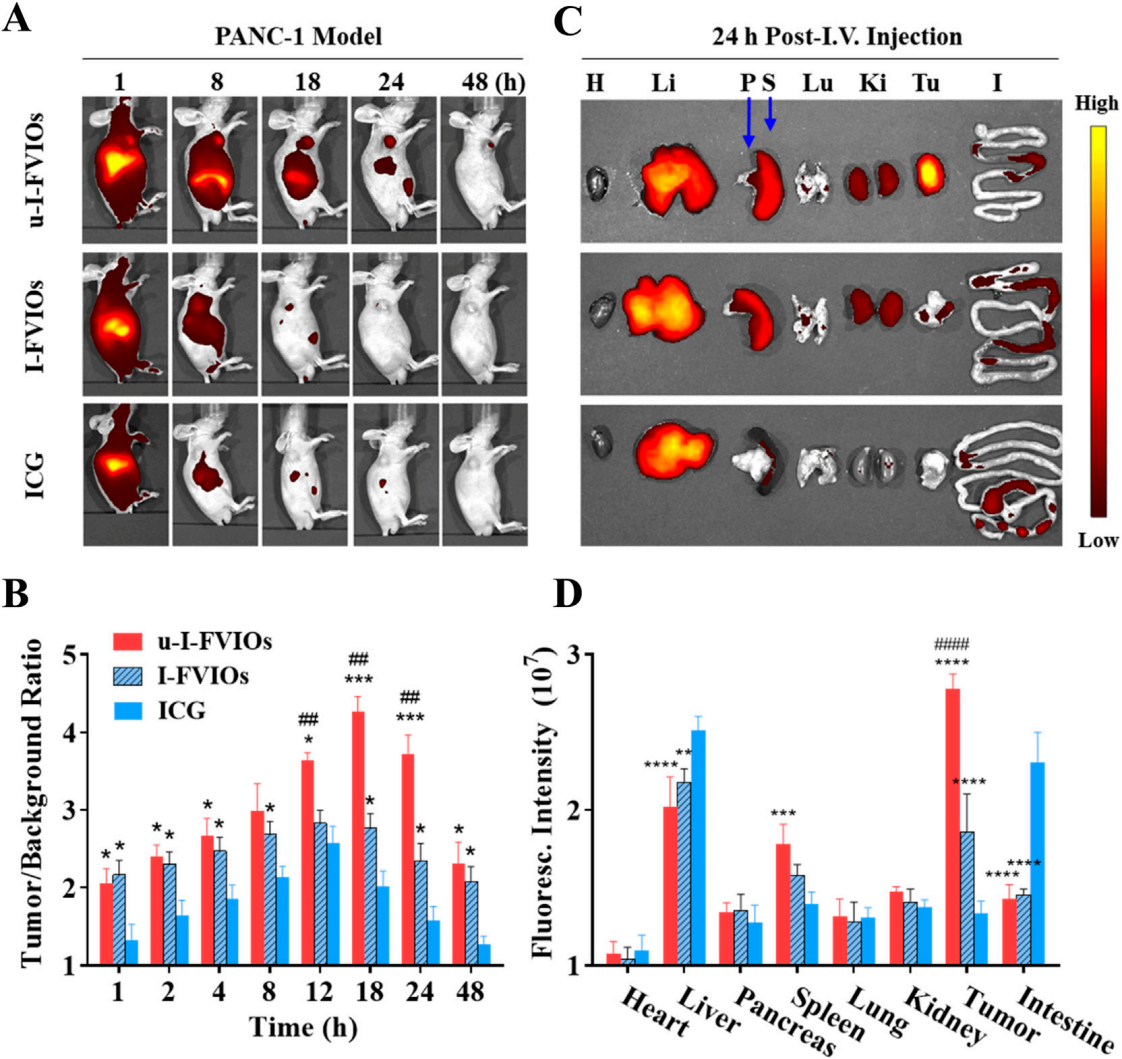
Biodistribution of u-I-FVIOs in mice post (I)V. injection. **(A)** Fluorescence Imaging and **(B)** Tumor-to-background ratios monitored over 48 h in PANC-1 subcutaneous tumor-bearing mice following (I)V. injection of 200 μL ICG (100.2 μg/mL), I-FVIOs (300 μg/mL), or u-I-FVIOs (300 μg/mL). **(C)** *Ex vivo* fluorescence Imaging and **(D)** Quantified fluorescence intensity of major organs and tumors were obtained 24 h post-injection. (H) heart; Li: liver; P: pancreas; S: spleen; Lu: lung; (K) kidney; T: tumor; (I) intestine. Compared to ICG, *: P < 0.05, **: *P* < 0.01, ***: *P* < 0.001, ****: *P* < 0.0001; compared to I-FVIOs, ##: *P* < 0.01, ####: *P* < 0.0001.

As shown in Figure 5A, all three groups exhibited strong NIR fluorescence at 1 h, which diminished almost completely by 48 h, except in the tumor region of the u-I-FVIOs group. The fluorescence signal decayed most rapidly in the ICG group, followed by I-FVIOs and u-I-FVIOs. Unlike the ICG group, in which no tumor-specific signal was observed, both the I-FVIOs and u-I-FVIOs groups displayed tumor-specific fluorescence retention up to 24 h and 48 h, respectively. In Figure 5B, tumor-to-background ratios (TBRs)—calculated as the ratio of the average fluorescence intensity in the tumor area to that in the background area—were compared among the three groups. Both u-I-FVIOs and I-FVIOs exhibited significantly higher TBRs across all time points than ICG (except for u-I-FVIOs at 8 h). The differences between u-I-FVIOs and I-FVIOs were statistically significant only at 12–24 h (*P* < 0.01), during which the TBRs of u-I-FVIOs were 3.5–4.5, compared with 2.5–3.0 for I-FVIOs and 1.5–2.5 for ICG. Notably, while TBRs peaked at 12 h in the I-FVIOs and ICG groups, the peak occurred later, at 18 h, in the u-I-FVIOs group. The *P* values were obtained using two-way ANOVA followed by *post hoc* Tukey’s *post hoc* test for multiple comparisons with recommended correction.

To further assess the biodistribution of u-I-FVIOs, tumors and major organs were harvested 24 h post-injection for fluorescence analysis (Figures 5C,D). All three agents exhibited strong accumulation in the liver. Notably, ICG exhibited significantly higher fluorescence than both u-I-FVIOs and I-FVIOs in the liver (by 24% and 15%, *P* < 0.01) and intestines (by 61% and 58%, *P* < 0.0001). Conversely, u-I-FVIOs and I-FVIOs demonstrated higher signal intensity in the spleen compared with ICG (by 27% and 13%), with only u-I-FVIOs reached statistical significance (*P* < 0.001). Consistent with the *in vivo* imaging results, u-I-FVIOs demonstrated a 1.5-fold (*P* < 0.0001) and 2.1-fold (*P* < 0.0001) higher signal intensity in tumor relative to I-FVIOs and ICG, and I-FVIOs also exhibited 1.4-fold higher (*P* < 0.0001) signal intensity in tumor than ICG.

### 3.6 MRI and fluorescence imaging of pancreatic cancer *in vivo*

**FIGURE 6:**
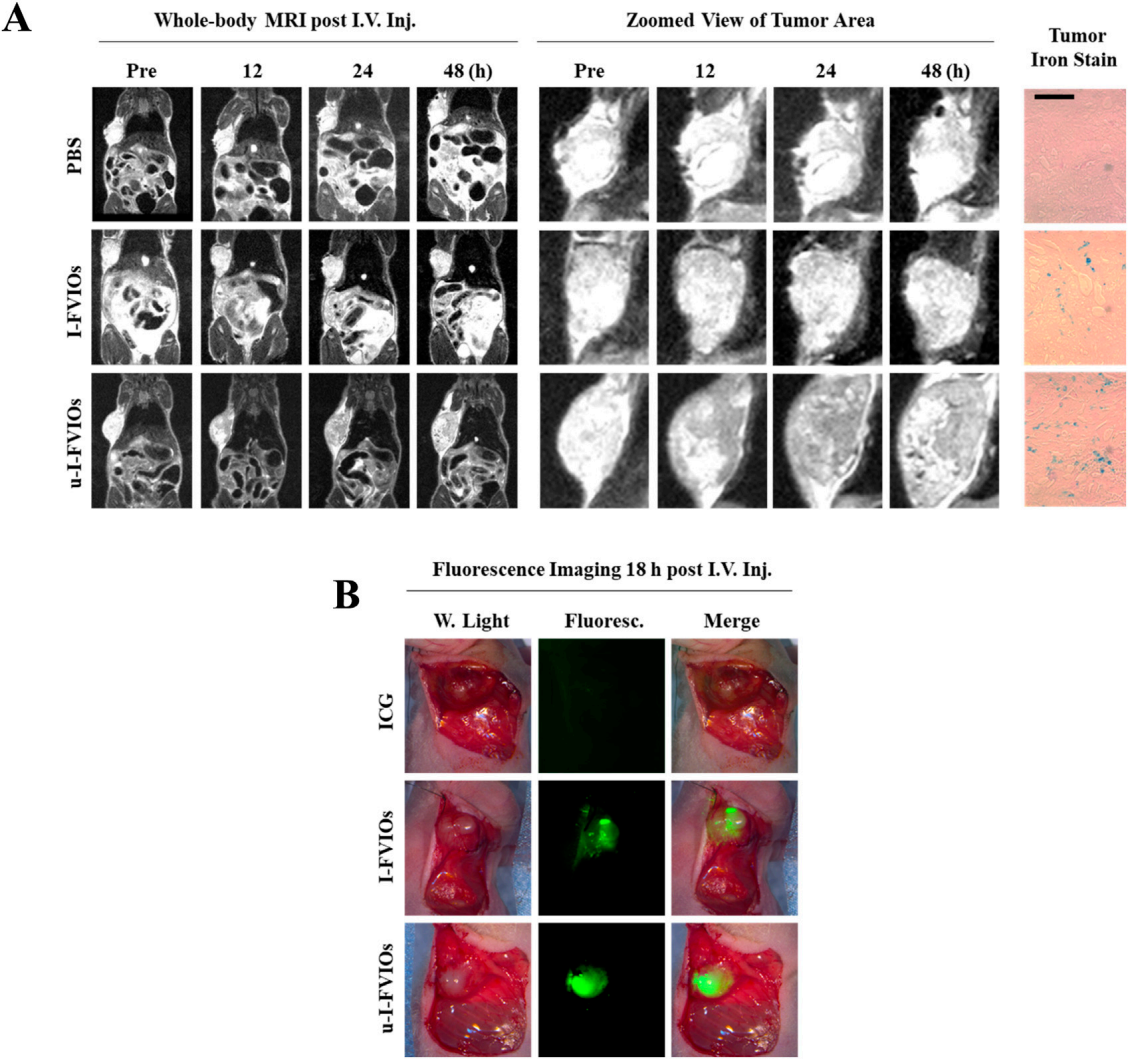
Dual-mode imaging capacity of u-I-FVIOs. **(A)** Whole-body MRI images and zoomed views of tumor area of PANC-1 tumor-bearing mice at baseline and at 12, 24, and 48 h after (I)V. injection of 200 μL of PBS, I-FVIOs (300 μg/mL), or u-I-FVIOs (300 μg/mL). Prussian blue staining of tumor sections at 24 h post-injection is also shown. Scale bar: 200 μm. **(B)** Fluorescence imaging for intraoperative identification of tumor regions in PANC-1 subcutaneous tumor-bearing mice18 h post-I.V. injection of 200 μL ICG (100.2 μg/mL), I-FVIOs (300 μg/mL), or u-I-FVIOs (300 μg/mL).

To evaluate the MRI capability of u-I-FVIOs *in vivo*, T2-weighted MRI was conducted to assess their accumulation and retention in tumors over time. As shown in Figure 6A, both the u-I-FVIOs and I-FVIOs groups exhibited significant signal darkening in the tumor region at 12 h post-injection. Notably, the u-I-FVIOs group showed more pronounced and prolonged signal reduction, which peaked at 24 h and persisted up to 48 h. As the MRI study did not include an 18 h time point, it is possible that the true signal peak, observed at 18 h in the fluorescence imaging study (Figure 5B), was missed. Prussian blue staining of tumor tissues harvested at 24 h post-injection further confirmed this result, revealing greater Fe-positive staining in the u-I-FVIOs group compared to the I-FVIOs group.

In a separate animal experiment, we assessed the feasibility of u-I-FVIOs-mediated intraoperative fluorescence imaging for more accurate tumor identification and excision. Eighteen hours after I.V. injection, the tumor region was clearly and sharply visualized with strong fluorescence. Although I-FVIOs also delineated the tumor, the margins were less distinct, and some areas appeared dimmer. In contrast, ICG failed to clearly differentiate tumor tissue from the surrounding normal tissue (Figure 6B).

### 3.7 *In vivo* magnetic hyperthermia therapy

**FIGURE 7:**
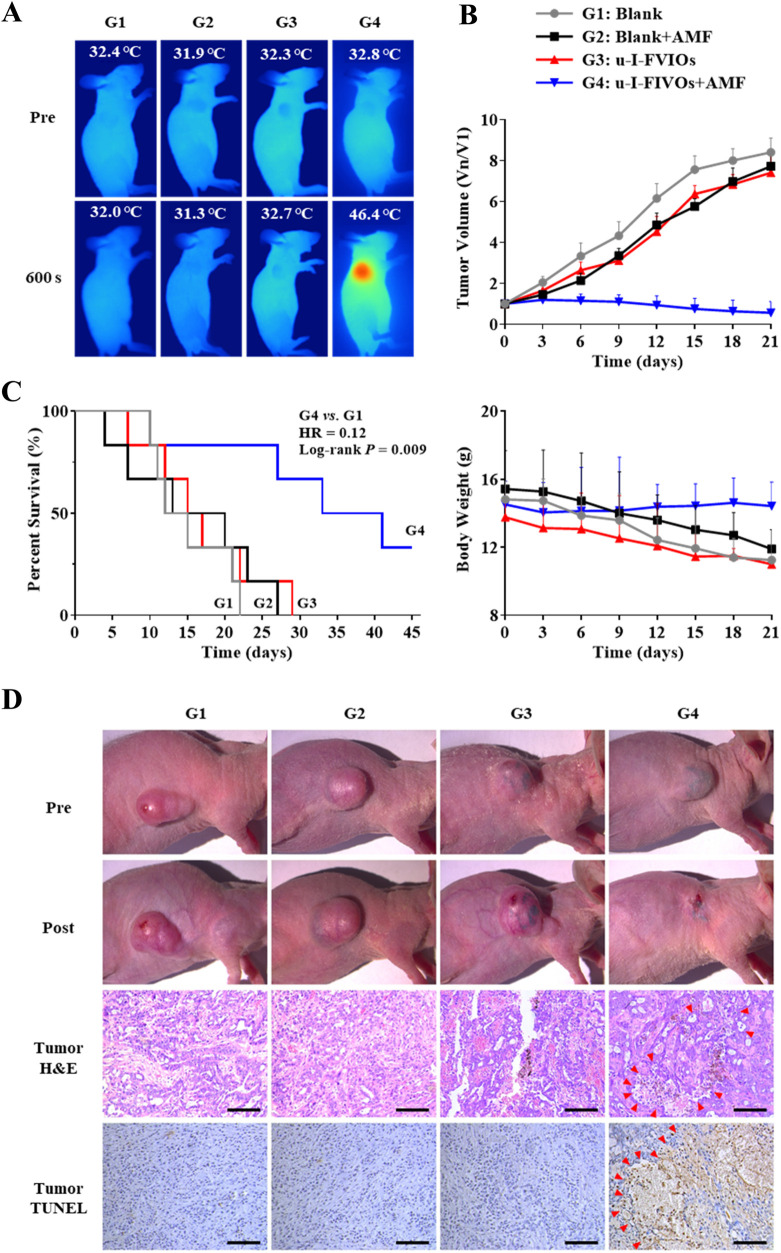
Efficacy and safety of magnetic hyperthermia therapy in PANC-1 subcutaneous tumor bearing mice. G1: PBS; G2: PBS + AMF (600 s on days 0 and 3); G3: u-I-FVIOs (1,000 μg/mL, 200 μL, (I)T., day 0); G4: u-I-FVIOs (1,000 μg/mL, 200 μL, (I)T., day 0) + AMF (600 s on days 0 and 3). **(A)** NIR thermal images captured before and after 600 s of AMF exposure in each group. **(B)** Changes in relative tumor volume (normalized to day 0) and body weight over 21 days of observation period (n = 6 per group). **(C)** Kaplan–Meier survival curves for each group (n = 6 per group), HR: hazard ratio. **(D)** Representative images of tumors before and after 2nd AMF treatment (images captured at different time points due to variations in animal survival times). Hematoxylin and eosin (H and E) staining and TUNEL staining of excised tumor tissues were performed 24 h after the 2nd AMF treatment. Arrows indicate necrotic and apoptotic regions within the tumor tissue. Scale bar: 100 μm.

For magnetic hyperthermia therapy (MHT), u-I-FVIOs or PBS was injected intratumorally (I.T.) into PANC-1 subcutaneous tumor-bearing mice. The mice were then exposed to an AMF or kept as controls for 600 s on days 0 and 3. As monitored by infrared thermal imaging, the tumor temperature in the u-I-FVIOs + AMF group rose to 46.4 °C—a level sufficient to induce effective tumor cell death. In contrast, no significant temperature elevation was observed in the other groups (Figure 7A). As shown in Figure 7B 21 days after initiation of MHT, the u-I-FVIOs + AMF group exhibited dramatic tumor shrinkage in all treated animals, with an average tumor suppression rate of 93% (*P* = 0.0001), while the other treatment groups showed no discernible anti-tumor effects. Mice in the u-I-FVIOs + AMF group tolerated the treatment well, with no significant change in body weight. In comparison, the other groups experienced slight weight loss, despite continued tumor growth, although the differences were not statistically significant. Histological analysis further confirmed the safety of magnetic hyperthermia, with no pathological changes observed in major organs (Supplementary Figure S2). In terms of survival, all mice in the control groups died within 30 days due to rapid disease progression. In contrast, only two mice in the u-I-FVIOs + AMF group died within 30 days, and two mice remained alive at the end of the study (Figure 7C), the hazard ratio (95% confidence intervals) for u-I-FVIOs + AMF group was 0.12 (0.02–0.59); Log-rank rest, *P* = 0.009. Additionally, histological examination using H&E and TUNEL staining revealed pronounced cellular damage in the u-I-FVIOs + AMF group (Figure 7D), including pyknosis, apoptosis, and coagulative necrosis, indicating strong localized cytotoxic effects. These findings were absent in the other groups.

## 4 Discussion

This study presents the first report on uPAR-ICG-FVIOs (u-I-FVIOs). Although uPAR, ICG, and FVIOs have each been individually investigated for diagnostic or therapeutic purposes, their integration into a single multifunctional nanomedicine has not been explored. In this system, uPAR facilitates cancer-specific targeting and enhances cellular uptake; ICG and FVIOs provide complementary dual-mode imaging capabilities (fluorescence and MRI); and FVIOs function as the core machinery enabling efficient AMF-induced heat conversion for magnetic hyperthermia therapy (MHT). While similar multifunctional strategies have been reported: for example, PD-L1-targeted dual-imaging probes (Liu et al., 2023) and TAT peptide-based MHT platforms (Fang et al., 2021), our u-I-FVIOs represent the first targeted nanomedicine to combine dual-mode imaging with MHT for pancreatic cancer.

uPAR is a well-established tumor-associated antigen (TAA). However, drugs targeting uPAR have not yet achieved commercial success, likely because uPAR functions as a non-therapeutic TAA—direct targeting does not elicit obvious anti-cancer effects. By contrast, most commercialization efforts have focused on therapeutic TAAs involved in signaling pathways essential for cancer cell survival or proliferation, such as HER2 and PD-1. However, recent advances in antibody conjugation technologies have notably accelerated the development of drugs that target non-therapeutic TAAs, such as PSMA (Fallah et al., 2023) and Trop-2 (Liu et al., 2024), which primarily serve as “homing mechanisms”. These non-therapeutic TAAs are particularly advantageous for imaging applications, due to their minimal or negligible systemic toxicity. As proof of clinical validity, two uPAR-targeted cancer imaging agents—one for PET/CT (Carlsen et al., 2022) and one for NIR (Andersen et al., 2025)—have already advanced to phase II clinical trials. In addition to imaging, uPAR-targeting strategy has been employed in the development of a wide range of novel cancer therapies, including oncolytic viruses, CAR-T cell therapies, and antibody-drug conjugates (Metrangolo et al., 2021).

The mechanisms underlying the retention of uPAR-targeting nanoparticles are multifaceted, as previously discussed by Yang et al. (2009): (i) retention is initiated through active binding of uPAR ligands to uPAR expressed on cancer cells and cancer stromal cells, followed by transcytosis; (ii) uPAR conjugation is essential for enabling nanoparticles to achieve tumor retention. In turn, once uPAR is conjugated to the nanoparticles, the resulting conjugates exhibit markedly longer residence times compared with uPAR-targeted peptides; (iii) passive distribution via leaky tumor vasculature is also thought to facilitate cancer-specific retention. Although the present study did not directly investigate the mechanisms of u-I-FVIOs retention, our cancer uptake assay (Figure 3C), fluorescence imaging (Figure 5), and MRI/iron staining (Figure 6A) consistently demonstrated superior tumor cell retention of u-I-FVIOs compared to FVIOs, and this retention appeared to be reversible.

In the fluorescence imaging studies, a single I.V. dose of u-I-FVIOs produced peak tumor-to-background signal ratios (TBRs) greater than 4 in all three animals at 18 h post-injection, substantially higher than those achieved in the ICG and I-FVIOs groups. A TBR of 4 is considered very high, as a systematic review of nearly 30 NIR fluorescent probes reported typical peak TBRs ranging from 2 to 3.8 (Neijenhuis et al., 2022). This suggests an optimal imaging time window (Figure 5B), and a markedly improved ability to distinguish tumor tissue compared to ICG-based imaging (Figure 6B), which could significantly enhance the accuracy of real-time intraoperative tumor detection. The enhanced contrast can be attributed to the markedly higher uptake of u-I-FVIOs by tumor cells (1.8- and 1.7-fold higher for PANC-1 and SW 1990) compared to normal cells observed *in vitro* (Figure 3C). This preferential uptake also accounted for the noticeably improved T2-weighted MRI performance: following I.V. injection of u-I-FVIOs, but not I-FVIOs, the MRI signals in tumor tissue exhibited a marked time-dependent reduction in T2 signal intensity, in stark contrast to the signals observed in healthy tissue (Figure 6A).

In the MHT animal study, exposure to an AMF for 600 s, following an I.T. injection of high-dose u-I-FVIOs, induced tumor hyperthermia, reaching 46.4 °C. I.T. administration was selected over I.V. injection based on both safety and clinical feasibility. I.T. delivery minimizes potential systemic toxicity, and can be readily implemented as a local ablation strategy for unresectable pancreatic tumors during laparoscopic surgery, particularly when guided by u-I-FVIOs fluorescence imaging for tumor margin identification. Importantly, approximately 70% of locally advanced pancreatic cancers remain unresectable despite FOLFIRINOX-based induction therapy, highlighting the growing role of local ablation as a complementary therapeutic option (Heger and Hackert, 2021). Although we did not directly measure the drug concentration in the tumors following the I.T. injection, the concentration-temperature relationship established *in vitro* suggested that the minimum tumor concentration required to achieve the temperature would be 50 μg/mL (Figure 2I).

The distinct anti-cancer effects of u-I-FVIOs MHT can be attributed to two main factors. First, the excellent energy-conversion efficiency of the FVIOs has been critical. It was reported that FVIOs can achieve a high specific absorption rate (SAR) exceeding 2000 W/g, whereas the SAR of clinically-approved SPIONs is less than one-tenth of that value (Liu et al., 2015; Tang et al., 2024). For MHT nanoparticles, a higher SAR is generally desirable, as it reflects greater efficiency in converting AMF energy into heat. However, a high SAR is not always advantageous, as it can be influenced by factors such as particle size, ambient temperature, and AMF strength. When a high SAR is achieved by increasing the AMF strength (frequency × amplitude) beyond the Hergt biological safety limit (∼5 × 10^9^ A m^-1^·s^-1^), it may pose a significant safety risk to the human body (Rodrigo et al., 2020). The AMF strength used in the present study (495 kHz, 220 Oe) was also adopted by previous studies, which was considered safe for the experimental animals (Gao et al., 2019; Tang et al., 2024). Second, high tumor uptake is likely a key determinant. Although I-FVIOs and u-I-FVIOs demonstrated comparable MHT capacity at the same concentration *in vitro* (Figure 2G), both the *in vitro* uptake experiment (Figure 3C) and *in vivo* imaging study (Figure 5) showed that tumor uptake of u-I-FVIOs was ∼1.5-fold higher than that of I-FVIOs. This ∼50% uptake difference may have led to tumor temperatures more than 10 °C lower when treated with the non-targeting FVIO agent, thereby falling short of the optimal therapeutic range of 43 °C–47 °C required for effective cancer cell killing (Imashiro et al., 2021).

The mechanisms underlying the temperature-dependent cancer killing are increasingly understood: mild hyperthermia (41 °C–45 °C) promotes apoptosis (Li et al., 2020), while higher temperature (>48 °C) causes immediate necrosis (Diederich, 2005). Beyond direct thermal ablation leading to necrosis and apoptosis, ferroptosis is also expected to contribute to antitumor efficacy of u-I-FVIOs, which act as an intrinsic iron source by releasing Fe^2+^/Fe^3+^ ions within the tumor microenvironment. Under AMF exposure, the localized heat drives the Fenton reaction, producing reactive oxygen species (ROS), lipid hydroperoxides (LPO), and glutathione peroxidase 4 (GPX4) (Tang et al., 2024). In addition, heat-ablated cancer cells release damage-associated molecular patterns (DAMPs) that activate tumor-associated dendritic cells and macrophages (TADCs and TAMs), thereby enhancing antitumor immunity and supporting recent advances in combining MHT with immunotherapies (Yang et al., 2025; Yuan et al., 2025).

From a safety perspective, ICG is an FDA-approved compound, and the safety of systemically administered FVIOs, at a much higher does than the current study, has been indicated previously in multiple animal experiments (Tang et al., 2024). In our *in vitro* cytotoxicity assays, u-I-FVIOs alone showed no detectable effect on cancer cell viability, even at concentrations much higher than those used for I.V. administration *in vivo*. Moreover, because normal cells generally lack uPAR expression, any cytotoxicity following u-I-FVIOs internalization is expected to be minimal in normal cells. In animal studies, I.T. injections of high-concentration u-I-FVIOs caused no significant changes in body weight or pathological alterations in major organs. Fluorescence and MRI imaging over 48 h suggested reversible u-I-FVIOs retention *in vivo*, while fluorescence imaging at 24 h post-I.V. injection suggests that both I-FVIOs and u-I-FVIOs undergo hepatic metabolism and renal excretion. This clearance profile appears distinct from that of ICG, which relies predominantly on hepatic metabolism and biliary excretion. Nevertheless, for future clinical trials, good laboratory practice (GLP)-compliant animal studies will be necessary to assess the long-term (3-month) safety and the effects of repeated administration of u-I-FVIOs on overall animal health and organ function.

Our study has several limitations. (i) As a preliminary study, some *in vitro* experiment conditions were less optimized, e.g., the u-I-FVIOs concentrations used in some *in vitro* studies were relatively low in consideration of their IC_50_ value. (ii) Developing orthotopic tumor-bearing animal models is particularly challenging due to the technical complexity of tumor transplantation surgery, the need for intensive post-operative care, the highly heterogeneous tumor microenvironment (TME) of pancreatic cancer, and the inherent variability in tumor growth and experimental timelines (Stribbling et al., 2024). Therefore, in this initial proof-of-concept study, we employed a subcutaneous xenograft tumor model, which is more easily established and provides greater experimental homogeneity. However, the subcutaneous pancreatic cancer model differs substantially from human pancreatic cancer in anatomy, physiology, and immunology. Building on the success of this study, future work will consider an orthotopic pancreatic cancer model to achieve closer anatomical and physiological relevance to the human disease (Lee et al., 2025). (iii) Although I.T. administration is clinical feasible, a broader application of u-I-FVIOs in cancer treatment should consider I.V delivery, as several systemic MHT approaches had been under investigation (Soleymani et al., 2020; Albarqi et al., 2020). The absence of a quantified I.V. dose–tumor concentration relationship in the present study limits the ability to predict the optimal I.V. dose required to achieve effective MHT, particularly given the narrow therapeutic temperature window and the need for high precision in targeting tumor concentrations. On the positive side, the tumor-targeting capability and T_max_ of I.V.-administered u-I-FVIOs were clearly demonstrated in the imaging studies (Figures 5, 6). Future studies should therefore aim to establish the dose–concentration relationship for temperature modeling and evaluate alternative systemic delivery routes, such as intraperitoneal injection.

## 5 Conclusion

In summary, we designed plasminogen activator receptor (uPAR)-targeted indocyanine green (ICG)–conjugated ferrimagnetic vortex-domain nanorings (u-I-FVIOs) for *in vivo* pancreatic cancer imaging and magnetic hyperthermia therapy (MHT). The synthesized u-I-FVIOs exhibited high biocompatibility, over 48 h serum stability, linear imaging capacity, strong cancer targeting capacity. Animal studies confirmed that u-I-FVIOs can serve as a dual-mode imaging platform for MRI and NIR fluorescence imaging, the latter offering great potential for image-guided surgery. u-I-FVIOs also demonstrated an AMF-dependent magnetic hyperthermia effect and high energy conversion efficiency. MHT with u-I-FVIOs resulted in over 90% tumor suppression and over 8-fold significantly prolonged survival in the pancreatic cancer animal model. In brief, our newly developed nanomedicine, u-I-FVIOs, shows great potential for imaging and MHT for clinical translation.

## Data Availability

The raw data supporting the conclusions of this article will be made available by the authors, without undue reservation.

## Supplementary material

SUPPLEMENTARY FIGURE S1 Distribution of size of u-I-FVIOs nanorings.Polydispersity index (PDI) < 0.2 is generally considered monodisperse and acceptable nanoparticle formulation.

SUPPLEMENTARY FIGURE S2 Hematoxylin and eosin (H&E) staining of major organs from subcutaneous PANC-1 tumor-bearing mice, collected 24 h after the 2nd AMF treatment in the magnetic hyperthermia therapy (MHT) experiment. Scale bar: 100 µm.
