# Supplementary figures and images for: Preclinical evaluation of uPAR-ICG-FVIOs for dual-mode imaging and magnetic hyperthermia therapy in pancreatic cancer
Source: Front Pharmacol. 2025 Nov 28;16:1681718. doi: 10.3389/fphar.2025.1681718 (PMC12699158; doi:10.3389/fphar.2025.1681718)

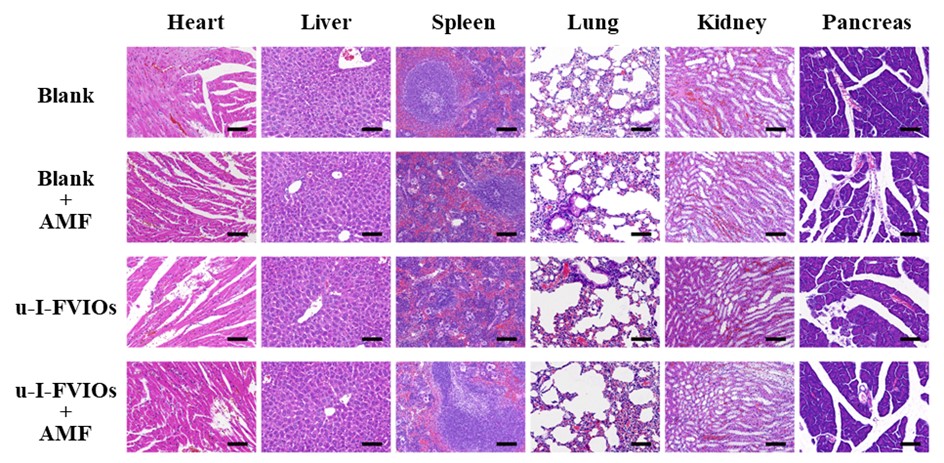

Supplement: Supplementary file 1 [file Image2.jpg]

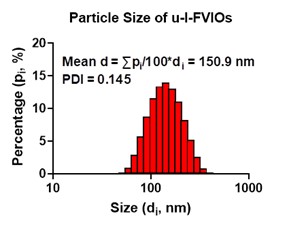

Supplement: Supplementary file 2 [file Image1.jpg]
